# Multiple functionally divergent and conserved copies of alpha tubulin in bdelloid rotifers

**DOI:** 10.1186/1471-2148-12-148

**Published:** 2012-08-17

**Authors:** Isobel Eyres, Eftychios Frangedakis, Elisabeth A Herniou, Chiara Boschetti, Adrian Carr, Gos Micklem, Alan Tunnacliffe, Timothy G Barraclough

**Affiliations:** 1 Department of Life Sciences, Imperial College London, Silwood Park Campus, Ascot, Berkshire, SL5 7PY, UK; 2 Present address: Department of Plant Sciences, University of Oxford, South Parks Road, Oxford, OX1 3RB, UK; 3 Institut de Recherche sur la Biologie de l’Insecte, CNRS UMR 7261, Université François-Rabelais, 37200, Tours, France; 4 Department of Chemical Engineering and Biotechnology, University of Cambridge, New Museums Site, Pembroke Street, Cambridge, CB2 3RA, UK; 5 Department of Genetics, University of Cambridge, Downing Street, Cambridge, CB2 3EH, United Kingdom; 6 Current address: Institute of Ecosystem Study, National Research Council, Largo Tonolli 50, 28922, Verbania Pallanza, Italy

**Keywords:** Bdelloid rotifers, Gene copies, Tubulin, Evolution

## Abstract

**Background:**

Bdelloid rotifers are microscopic animals that have apparently survived without sex for millions of years and are able to survive desiccation at all life stages through a process called anhydrobiosis. Both of these characteristics are believed to have played a role in shaping several unusual features of bdelloid genomes discovered in recent years. Studies into the impact of asexuality and anhydrobiosis on bdelloid genomes have focused on understanding gene copy number. Here we investigate copy number and sequence divergence in alpha tubulin. Alpha tubulin is conserved and normally present in low copy numbers in animals, but multiplication of alpha tubulin copies has occurred in animals adapted to extreme environments, such as cold-adapted Antarctic fish. Using cloning and sequencing we compared alpha tubulin copy variation in four species of bdelloid rotifers and four species of monogonont rotifers, which are facultatively sexual and cannot survive desiccation as adults. Results were verified using transcriptome data from one bdelloid species, *Adineta ricciae*.

**Results:**

In common with the typical pattern for animals, monogonont rotifers contain either one or two copies of alpha tubulin, but bdelloid species contain between 11 and 13 different copies, distributed across five classes. Approximately half of the copies form a highly conserved group that vary by only 1.1% amino acid pairwise divergence with each other and with the monogonont copies. The other copies have divergent amino acid sequences that evolved significantly faster between classes than within them, relative to synonymous changes, and vary in predicted biochemical properties. Copies of each class were expressed under the laboratory conditions used to construct the transcriptome.

**Conclusions:**

Our findings are consistent with recent evidence that bdelloids are degenerate tetraploids and that functional divergence of ancestral copies of genes has occurred, but show how further duplication events in the ancestor of bdelloids led to proliferation in both conserved and functionally divergent copies of this gene.

## Background

Bdelloid rotifers are microscopic aquatic invertebrates of particular interest for two features of their lifestyles. First, they have persisted for tens of millions of years [1] and diversified into nearly 450 recognized species [2], despite the apparent absence of meiosis and sex [3]. Second, adult bdelloids can survive essentially complete desiccation in a state known as anhydrobiosis [4]. Moreover, bdelloid genomes show a number of unusual features that might relate to their long-term asexuality and ability to survive desiccation [5]. Understanding these features can shed light not only on bdelloids’ unusual lifestyle, but also on the range of mechanisms by which animal genomes can evolve.

In efforts to understand bdelloid genome organization, much interest has focused on gene copy numbers. The first work on bdelloid genomes found that bdelloids possessed divergent copies of genes present as a single diploid locus in their closest sexual relatives, the monogonont rotifers [6]. It was hypothesized that these were former alleles that had diverged following the loss of meiosis and recombination [7], a process called the Meselson effect [8]. Later work suggested that putative former alleles of LEA (Late Embryogenesis Abundant) protein had diverged so much as to adopt different biochemical roles protecting bdelloid cells from desiccation [9]. Although functional divergence of gene copies is not restricted to asexual lineages, it was argued that functional divergence of former alleles might have contributed towards adaptation to desiccating environments.

Subsequent work has shown that bdelloid genome complexity cannot be explained simply through divergence of former diploid alleles: bdelloids are degenerate tetraploids [10,11]. Sequencing the genomic regions surrounding four copies of hsp82 revealed that the chromosomes comprise two collinear pairs. Between pairs, divergence is so high that they only share a handful of genes, whereas within collinear pairs divergence is much lower and there are regions of complete identity. The same pattern was found in other genomic regions and in different bdelloid families [12]. It was proposed that bdelloids use pairs of homologous chromosomes as templates to repair DNA breaks caused during desiccation, and in the process sequences are homogenised at broken regions by gene conversion [5,10]. This provides an explanation for the patchy nature of divergence within collinear pairs and for bdelloids’ uncommon ability to repair DNA breaks caused by desiccation and ionizing radiation [13]. The original cause of tetraploidy is unknown but could have involved either a genome-wide duplication event or hybridization between sexual ancestors – the latter being commonly associated with asexuality in many organisms [14].

Together, these findings make bdelloids an interesting case study for understanding opposing forces on gene copy evolution. Although divergent copies of bdelloid genes can no longer be interpreted simply as former alleles, it remains of interest to know how often divergent copies of genes have been maintained and diverged in function. In principle, the maintenance of functionally divergent copies of genes could provide bdelloids with an enhanced genetic repertoire and, perhaps, increased phenotypic plasticity [9].

Here, we explore copy evolution in alpha tubulin and ask whether divergent copies have been maintained in bdelloid genomes. Alpha tubulin forms microtubules with beta tubulin and provides a range of functions in cellular movement and cell division. Previous studies of copy variation of alpha tubulin in other organisms revealed contrasting patterns making it an interesting gene to study in bdelloids [15]. In many animals, it is present in low copy number and highly conserved, which has led to its use as a marker for deep phylogenetic relationships [16]. However, the different roles of microtubules have led to functional divergence in many cases. Four distinct copies are present in *Drosophila melanogaster* and expressed in different tissues: including ovary and testis-specific forms [17,18]. If there were male-function or meiosis specific copies in sexual rotifers [19,20], bdelloids might be expected to have lost those copies or for them to have evolved new functions. Duplication and diversification of tubulin genes has also been implicated in adaptation to extreme environments in the Antarctic fish *Notothenia coriiceps*[21], which expresses at least nine different alpha tubulin isotypes that vary in stability and polymerization rates at extremes of temperature [22]. By expressing copies stable at different temperatures, function is maintained despite variation in environmental conditions. In plant seedlings alpha tubulin is up-regulated during both dehydration and rehydration [23-25]. Therefore, bdelloids might make use of their ancestral tetraploid state and maintain alpha tubulin copies to perform specialist functions during cycles of desiccation [9].

We used cloning and preliminary whole transcriptome data to characterize alpha tubulin copies in several monogonont and bdelloid rotifer species. Monogononts contained either one or two copies with very similar amino acid sequence (each presumably representing a single diploid locus). Contrary to the four or eight copies expected if bdelloids retained copies from their tetraploid ancestor, we found between 11 and 13 copies in each of four species of bdelloids. Phylogenetic relationships indicate that multiple copies were inherited from the common ancestor of bdelloids and some of them were lost or homogenized in some species. Approximately half of the copies retained amino acid sequences similar to the monogonont copies, whereas the rest have diverged into four distinct classes that differ in intron structure and biochemical properties predicted by computational analysis. Distinct classes have diverged significantly in amino acid sequence relative to the purifying selection observed within classes. The findings do not contradict current understanding of bdelloid genome organization, but show how further duplication events in the ancestor of bdelloids led to proliferation in both conserved and functionally divergent copies of this gene. The possible functional significance of alpha tubulin diversity is discussed in relation to sequence characteristics.

## Methods

### Study species, DNA extraction, cloning and sequencing

Four species of bdelloid rotifer belonging to two bdelloid families were compared to four species of monogonont rotifers also from two families (Table 1). Sequences for one of the monogononts, *Brachionus plicatilis,* were taken from the cDNA library of Suga *et al.*[26]. For the other monogonont species DNA was extracted from wild-caught individuals using Chelex InstaGene Matrix (Bio-Rad) following the manufacturers instructions. Genomic DNA from bdelloid species was extracted using the DNeasy blood and tissue kit (Qiagen). For each bdelloid species DNA was extracted from ~50 individuals from populations grown from a single female. Extractions were performed according to manufacturers instructions, and samples were heated at 56°C for 1 h for protein digestion. DNA quality was checked on a 1% agarose gel. PCR amplification was performed using 2 μl template DNA and Illustra PuRe-Taq Ready-To-Go PCR beads. Alpha tubulin was amplified using the primers TOA1F 5^′^-RGTNGGNAAYGCNTGYTGGGA-3^′^ and TOA1R 5^′^-CCATNCCYTCNCCNACRTACCA-3^′^[16], which amplify >90% of the coding region of each gene [27]. The amplification profile consisted of an initial denaturation of 5 min at 94°C, followed by 35 cycles of 45 s at 94°C, 60s at 58°C and 60s at 72°C and a final extension of 5 min at 72°C. Products of expected size (~1500 bp – 1800 bp) were cloned using the TOPO TA cloning kit (Invitrogen) and alpha tubulin inserts were re-amplified from individual clones. Amplicons were sequenced in both directions using the BigDye Terminator v3.1 Cycle Sequencing Kit (Applied Biosystems) and an ABI 3130 Genetic Analyzer (Applied Biosystems). Sequence data are available in GenBank (accession numbers: JX193018 – JX193069).

**Table 1:** Rotifer samples used in the study

**Species**	**Taxonomy (Order/Family)**	**Sample location**	**Collector/ Publication**	**Date collected/ time propagated in lab**	**Habitat**
*Machrotrachela quadricornifera* Milne, 1886	Bdelloidea Philodinidae	Fontaneto d’Agogna, Italy 45.6306, 8.4536	Diego Fontaneto	05-03-2006 3 months, to a population of ~100 individuals	Submerged moss in a stream, running water.
*Philodina flaviceps* Bryce, 1906	Bdelloidea Philodinidae	Fontaneto d’Agogna, Italy 45.6260, 8.4432	Diego Fontaneto	01-05-2005 3 months, to a population of ~100 individuals	Stream, running water.
*Adineta vaga* Davis, 1873	Bdelloidea Adinetidae	Bergen, Norway 60.3955, 5.3513	Timothy Barraclough	06-2004 3 months, to a population of ~100 individuals	*Sphagnum* in pond.
*Adineta ricciae* Segers & Shiel, 2005	Bdelloidea Adinetidae	Ryan’s billabong, Victoria, Australia	Segers and Shiel 2005	26-06-1998	Dry pond (billabong).
				clonal population propagated for years	
		−36.1086, 146.9777			
*Brachionus plicatilis* Muller, 1786	Monogononta Brachionidae	Lab strain NH1L	Suga *et al.* 2007	Hagiwara *et al.* 1993 in Suga *et al.* 2007	Seawater
*Brachionus quadridentatus* Hermann, 1783	Monogononta Brachionidae	Japanese pond, Silwood Park, UK	Diego Fontaneto	30-05-2007	Plankton
				DNA extracted from a single animal	
		51.4074, -0.6405			
*Brachionus urceolaris* Muller, 1773	Monogononta Brachionidae	Japanese pond, Silwood Park, UK	Diego Fontaneto	30-05-2007	Plankton
				DNA extracted from a single animal	
		51.4074, -0.6405			
*Synchaeta pectinata* Ehrenberg, 1832	Monogononta Synchaetidae	Japanese pond, Silwood Park, UK	Diego Fontaneto	30-05-2007	Plankton
				DNA extracted from a single animal	
		51.4074, -0.6405			

### Sequence alignment

Sequences were confirmed to be alpha tubulin using BLASTN, and aligned in Geneious Pro 5.0.3 [28] using Muscle [29] followed by manual adjustments. Wise2 [30] was used to define introns, which were manually checked by comparison to alpha tubulin cDNA from the monogonont rotifer *Brachionus plicatilis* (GenBank: BJ999223.1). Sequences used in our analyses contain no premature stop codons. Taq polymerase has a baseline error rate of 1 in 9000 base pairs [31]. Normally, when sequencing from uncloned PCR products this error rate is negligible, as errors occur at random sites across the amplified products and are averaged out upon sequencing. However, cloning to separate different copies of alpha tubulin within each sample introduces greater errors into the sequencing process [32]. Based on the estimated error rate of Taq of 1 in 9000 and the 35 cycles in our PCR reaction, we estimated an overall error rate of 0.38% per sequence (= 35/9000), which we rounded to 0.5% to allow for any additional error in sequencing these long products. Consequently, sequences obtained from cloning were considered to be the same if they were <1% different, and collapsed to a single majority rule consensus sequence, thus providing us with a conservative estimate of copy number. Plots of the number of distinct copies against the number of clones sequenced were used to assess whether most copies present in the genome had been discovered. The Chao estimator was calculated to estimate the potential number of missing copies [33], based on the assumption that if a few copies have been sampled only once or twice, then it is likely that some copies remain unsampled.

### Phylogenetic analyses

Relationships among copies were inferred from exon sequences using Bayesian Markov Chain Monte Carlo (MCMC) analyses implemented in p4 (v0.88.r221; [34]). We used p4 because preliminary investigations revealed compositional heterogeneity in our sequences [see Additional file 1. Introns were excluded from phylogenetic analysis because their extreme variability in sequence and insertion site confounded the evolutionary signal. JmodelTest [35] selected the GTR (general time-reversible) model [36] with gamma-distributed rate variation and a proportion of invariable sites (GTR + I + G) as the most likely substitution model using the Akaike Information Criterion. A chi-squared test revealed significant compositional homogeneity over the tree (chi-sq = 1053.36, p < 0.0005), and comparison of the observed compositional heterogeneity among sequences to a null distribution simulated using the fitted GTR + I + G model, confirmed that composition of the observed sequences was significantly heterogeneous (10000 simulations, p < 0.00005). The GTR + I + G model was therefore compared to node-discrete composition heterogeneity models NDCH(2) and NDCH(5) [37], which incorporate two and five tree-heterogeneous base-composition vectors respectively [see Additional file 2. The preferred model was chosen using Bayes Factors: a log_*e*_(Bayes Factor) of 5 or more was considered as very strong support for improved fit of a more complex model [38]. Bayesian MCMC analysis was run with the chosen model and two independent runs of four chains each for 4000000 generations. Posterior probabilities were calculated for support.

A second tree was reconstructed, using maximum likelihood implemented in PHYML 2.4.5 [39], from the translated amino acid alignment. ProtTest 2.4 [40] selected the JTT model of amino acid substitution [41,42] with gamma-distributed rate variation. Statistical support was assessed using aLRT non-parametric branch support [43]. Trees were rooted on the branch connecting bdelloid and monogonont sequences.

### Tests for selection

We estimated the ratio of the rates of non-synonymous and synonymous substitutions (dN/dS ratio) using likelihood methods implemented in PAML version 4 [44]. First, we fitted a model with a single dN/dS ratio across all bdelloid copies of alpha tubulin. Second, to test for divergent selection acting on different classes of alpha tubulin, we fitted a model with a different dN/dS ratio on branches connecting different classes (defined based on intron structure and described in the Results) versus on branches connecting sequences of the same class [45]. Third, we fitted a branch-site model to test for the presence of codons showing purifying selection on within-class branches (dN/dS ratio < 1) but evolving either neutrally or by positive selection on between-class branches (dN/dS ratio ≥ 1) [46]. Bayes empirical Bayes (BEB) inference was used to identify codons belonging to this set. We predict that the branch-site model should be preferred if amino acid divergence between the different classes has evolved through selection for functional specialization of some parts but not all of the protein. The best model was selected using the Akaike Information Criterion. Models were run using the average nucleotide frequencies at each codon position to specify equilibrium codon frequencies (option CodonFreq = 2 in PAML). To check for sensitivity to the observed compositional heterogeneity, we repeated the analyses using free parameters for each codon frequency (CodonFreq = 3). All inferences from model results were qualitatively unchanged over the simpler versions.

### *Adineta ricciae* transcriptome searches

To further assess the completeness of sampling of copies by cloning and sequencing, and to establish if multiple copies were expressed under experimental conditions, we searched an expressed sequence tag (EST) library [47] and draft transcriptome [48] sequences of A. ricciae for alpha tubulin copies. The transcriptome was generated by paired-end sequencing of ~200bp cDNA fragments using Illumina parallel sequencing. The resulting 19.5 million 76 bp reads were assembled with the CLC-bio (www.clcbio.com) assembler to give a library of 61,219 transcripts of size range 118-3674 bp. More details are provided in [48]. The databases were searched by BLASTN [49] for matches to the sequences obtained by cloning (with introns removed).

### Protein chemical analyses

Three parameters indicative of predicted protein chemistry, and hence potentially of changes in protein function, were calculated for each alpha tubulin amino acid sequence using ProtParam [50,51].

i) The grand average of hydropathy (GRAVY) index indicates the hydropathy of proteins: a positive GRAVY score means the protein is hydrophobic and a negative score hydrophilic [52]. Detrich [22] hypothesized that increasing contact surface hydrophobicity might enhance polymer stabilizing interactions in microtubules, so a more hydrophobic (positive) GRAVY score could indicate a more stable microtubule.

ii) Isoelectric point (pI) is the pH at which a protein carries no net electrical charge, which can affect its solubility at a given pH. Zambito *et al.*[53] found a wide variation in the pI of rat brain tubulin isotypes, and showed that some variants are associated with particular cellular compartments. Variation in pI of our alpha tubulin copies may therefore imply variation in cellular function.

iii) The instability index provides an estimate of a protein’s folding stability *in vitro*, based on estimated instabilities of the protein’s dipeptides. A protein with an instability index of less than 40 is predicted as stable, a value above 40 predicts that the protein may be unstable, and therefore less viable as a functional protein.

Predictive discriminant analysis was performed using the predict.lda function of the MASS library version 7.2-48 [54] in the R Statistical Programming Language R 2.9.2 [55] to see if classes of copies based on intron structure are predicted by these three protein chemical properties, i.e. whether copies with different intron structure have divergent chemical properties.

Multivariate analysis of protein polymorphism (MAPP) scores of physiochemical impact were estimated for all codons [56], providing a measure of constraint at each site (a high mean MAPP score indicating that amino acid substitution would have large impact on function). To determine if sites that diverged significantly between classes had significantly different MAPP scores to the rest of the protein, the mean MAPP score at codons identified by the BEB analysis (posterior probability > 0.95) to be significantly divergent between classes (described in the *Tests for Selection* section above) was compared to 10000 replicates of the mean MAPP score at the same number of random sites drawn from a set of all sites in the protein. To determine if sites identified by the BEB analysis had significantly different MAPP scores to polymorphic sites not evolving faster between classes than within them, the mean MAPP score at the codons identified by the BEB analysis (posterior probability > 0.95) was compared to 10000 replicates of the mean MAPP score at the same number of random sites drawn from a set of all polymorphic sites.

### Protein structural analyses

Translated protein sequences were mapped to the structural and functional regions of alpha tubulin identified by [57]. Regions of alpha helices and beta sheets were classified as structural. Regions falling into the following four types were classified as functional: the interface with beta tubulin; the regions interacting with GTP (a single domain interacting with ribose, the three amino acids that interact with the nucleotide, and the phosphate interacting domains); the tubulin nonexchangeable site (GTP binding); and the C-terminal helices (H11 and H12) where MAPs (microtubule associated proteins) and motor proteins usually bind. To determine whether amino acid divergence was concentrated in particular structural regions, amino acid positions identified by the BEB analysis to be significantly divergent between classes were mapped onto the identified functional and structural regions. Monte Carlo tests were used to test for significant concentration of divergent amino acids either in structural or in functional regions of the genome.

## Results

### Number of copies of alpha tubulin genes from cloning and sequencing

**Figure 1:**
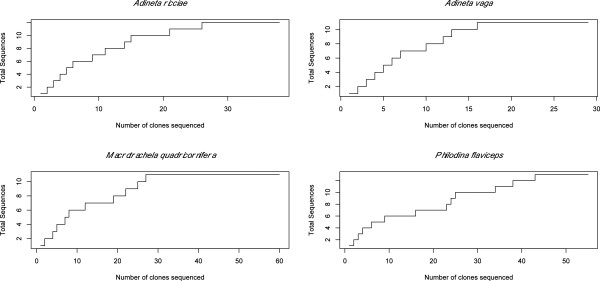
Saturation curves comparing sampling effort (number of clones sequenced) with number of unique sequences identified.

The *B. plicatilis* EST database of Suga *et al.*[26] contained two alpha tubulin copies (BJ999249 and BJ999223) that were 13% divergent at the nucleotide level but only differed in three amino acids. Amplifying and sequencing alpha tubulin in the three selected monogonont species (*B. urceolaris, B. quadridentatus* and *S. pectinata*) revealed just one copy of alpha tubulin in each species. Pairwise amino acid divergence between all monogonont alpha tubulin sequences was only 0.7%. Both *B. plicatilis* copies contained our primer binding sites and should therefore have been amplified using our primers, if they had been present in the other monogonont species. Cloning and sequencing of bdelloid alpha tubulin yielded between 11 and 13 different copies in each species (Table 2). This is a conservative estimate because sequences were only considered unique when they varied by >1% to account for 99.5% PCR fidelity of the cloned copies. Including all sequences obtained by cloning would increase the number of copies per species to almost as many as the number sequenced, but would not alter our broad findings beyond increasing the number of very similar copies within species [see Additional file 3. Saturation curves showed that the number of copies reached a plateau against the number of clones sequenced in each species (Figure 1). The Chao1 estimator indicated that all copies were likely to have been sampled in all species*.*

**Table 2:** Number of clones and unique sequences in bdelloid species

**Species**	**Total copies cloned**	**Unique sequences observed**	**Chao1 estimate of number of unique sequences (± standard error)**
*A. ricciae*	38	12	12.1 ± 0.3
*A. vaga*	29	11	11.1 ± 0.3
*M. quadricornifera*	60	11	11.0 ± 0.7
*P. flaviceps*	55	13	13.3 ± 0.7

### Intron and exon structure

**Figure 2:**
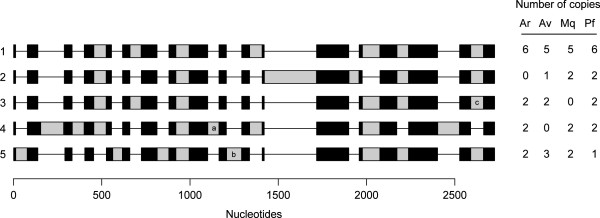
**Alignment of the five major copy types of alpha tubulin in bdelloid rotifers.** Classes based on intron presence/absence. Black block = exon, grey block = intron, line = gap. Copy classes 4, 5 and 3 contained variants with introns indicated by a, b and c respectively that were absent in copies otherwise sharing the same intron structure. Numbers of copies in each species are shown: *A. ricciae* (Ar), *A. vaga* (Av), *M. quadricornifera* (Mq) and *P. flaviceps* (Pf).

No introns were present in the monogonont sequences. In contrast, each bdelloid alpha tubulin copy contained between 6 and 8 introns located across 17 different sites (Figure 2). The copies fall into five major classes based on differences in intron content and location, with some additional losses or gains of single introns in particular species leading to a total of eight different intron patterns (Figure 2). Most introns were between 42 and 70 nucleotides in length (0 and 95 percentiles of intron lengths across all introns), but two introns were consistently longer across copies and species (intron 2: 107-131 bp, intron 12: 166-295 bp). All introns present in two or more classes were always found in class 1 and there were no unique introns in class 1. Class 3 only differed from class 1 by the loss of one intron (and a second intron in the *A. ricciae* sequences) whereas classes 2, 4 and 5 each contained one or more unique introns. All seven introns present in the class 1 copies shared a GGT motif at the 5’ splice site across all the classes. One intron unique to class 4 had the same 5’ splice site as the introns in class 1 copies and represents a putative translocation of an adjacent intron that is present in all the other classes but lacking in class 4. All other introns had different 5^′^ splice site motifs. Only minor changes in length of coding regions were observed among classes.

### Phylogenetic relationships among classes based on exon nucleotide sequences

**Figure 3:**
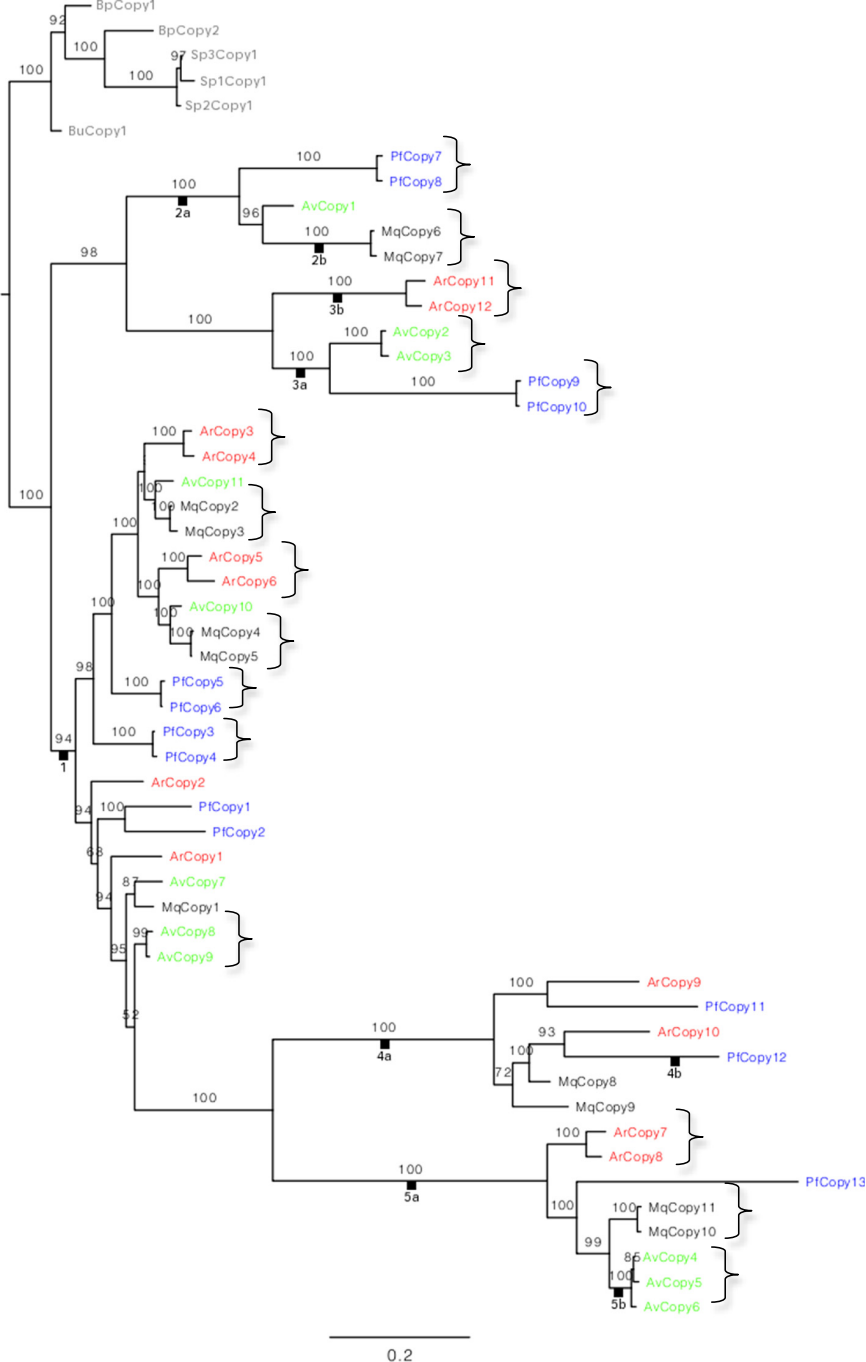
**Phylogenetic relationships among alpha tubulin copies based on bayesian analysis of the exon nucleotide alignment.** Classes defined by intron structure in Figure 2 are indicated by labels on branches. Species are indicated by colours: monogonont outgroups = grey; *A. ricciae* = red; *A. vaga* = green; *M. quadricornifera* = magenta; *P. flaviceps* = blue. Putative copies homogenized by gene conversion are indicated by curly brackets.

**Figure 4:**
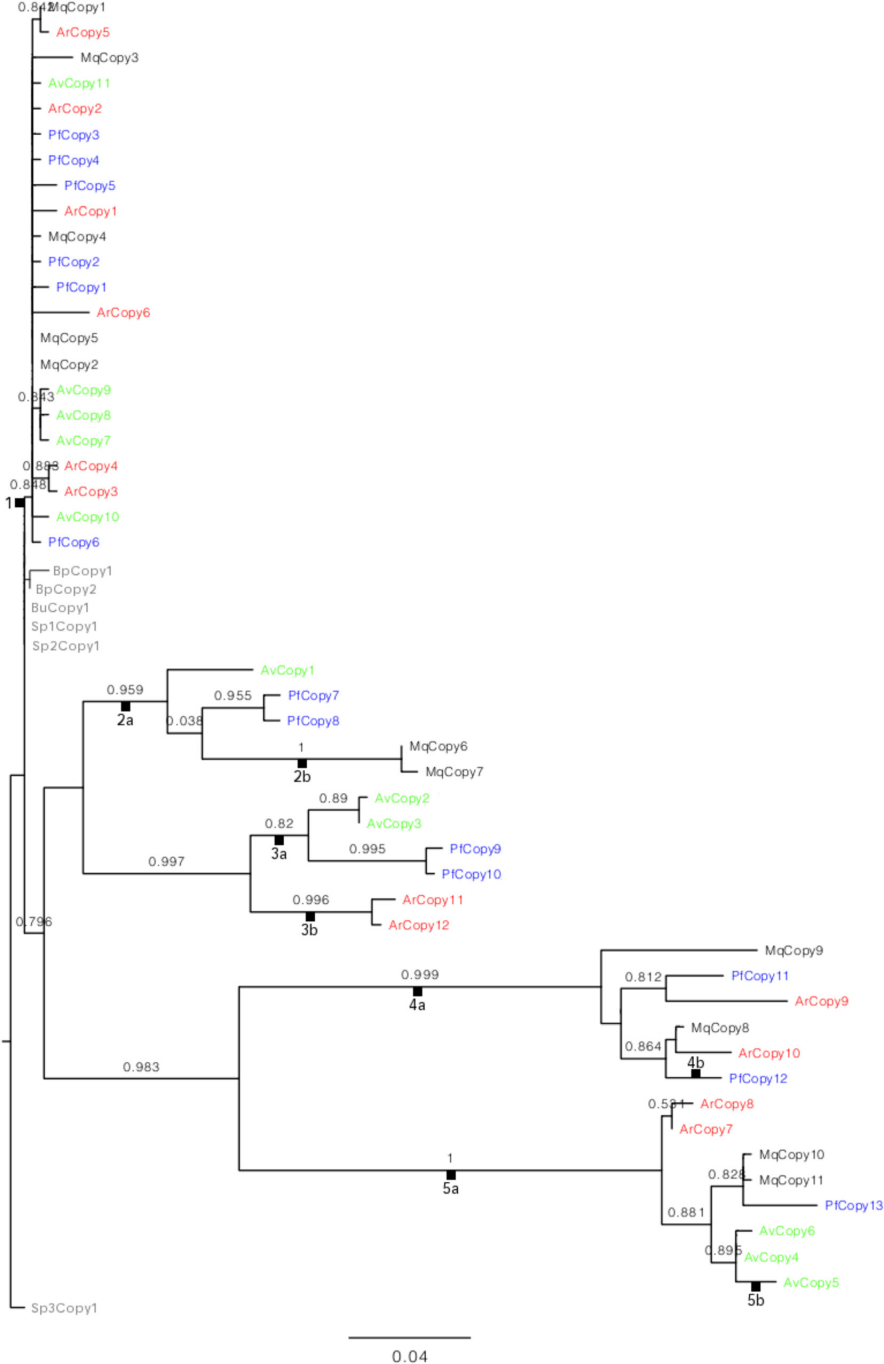
**Phylogenetic relationships among alpha tubulin copies based on maximum likelihood analysis of amino acid sequences.** Classes defined by intron structure in Figure 2 are indicated by labels on branches. Species are indicated by colours: monogonont outgroups = grey; *A. ricciae* = red; *A. vaga* = green; *M. quadricornifera* = magenta; *P. flaviceps* = blue.

Bdelloid alpha tubulin copies are monophyletic with respect to monogonont copies (posterior probability, pp = 1.0, Figure 3). The five classes of bdelloid copy types based on intron structure also form groups in the phylogenetic analysis of exon nucleotide sequences. Four classes (2–5) are monophyletic and supported by posterior probabilities of 1.0. Classes 2 and 3 are sister clades (pp = 0.98), and together form a monophyletic clade sister to all other bdelloid alpha tubulin sequences (pp = 1.0). Classes 4 and 5 are also sister clades (pp = 1.0). Their position relative to other sequences is unstable among different analyses: in the p4 analysis they apparently derive from within the paraphyletic grade of class 1 copies (pp =0.52), whereas in the amino acid tree they are sister clade to the class 2 and 3 clade (aLRT = 0.8, Figure 4). Class 1 copies are monophyletic (aLRT = 1.0) in the amino acid tree but paraphyletic in the nucleotide tree. All four species have class 1 and class 5 copies, whereas *A. ricciae* lacks class 2, *M. quadricornifera* lacks class 3, *P. flaviceps* lacks class 4 copies (Figure 2).

### Sequence divergence among copies

Across all classes, there were fifteen cases of pairs of very similar copies (<4.5% nucleotide sequence divergence) belonging to the same species (Figure 3). This is consistent with there being pairs of alpha tubulin copies homogenized by gene conversion as found for other bdelloid genes. One case of three similar sequences in *A. vaga* class 5 was also found and in this cluster, one copy has gained an intron lacking in the other two. Class 4 lacked any clusters of very similar sequences.

The groups of similar copies within species were always separated from their next closest sequence by >10% pairwise sequence divergence. We refer to copies differing by more than 10% sequence divergence as divergent copy types (note that each copy type sometimes contains two or three similar sequences belonging to the same species and <4.5% divergent as described in the preceding paragraph). Classes 2, 3 and 5 only contained one copy type of alpha tubulin per species (Figure 3). Class 1 copies included three (*A. vaga*) or four ( *A. ricciae, P. flaviceps* and *M. quadricornifera*) divergent copy types in each species and class 4 contained two copy types per species. The nearest divergent copy type to each sequence (or cluster of similar sequences) was always from a different species except for two pairs of divergent *P. flaviceps* class 1 copies. In all cases where both *A. vaga* and *A. ricciae* are present in a mini-species tree of copy types, contrary to classical taxonomy, *A. vaga* appears more closely related to *M. quadricornifera* than to *A. ricciae*.

### Amino acid variation and tests for selection

Comparison of the nucleotide and amino acid trees revealed high amino acid divergence between classes compared to nucleotide divergence between them (Figure 4): classes are separated by long amino acid branches relative to branch lengths within classes. This pattern is particularly striking in class 1, in which amino acid sequences differ by less than 1.1% whereas the nucleotide sequences have diverged to yield highly supported clades. Class 1 copies are similar to monogonont alpha tubulin copies (mean pairwise amino acid divergence between bdelloid and monogonont copies = 1.1%).

The branch-class model in PAML optimized a higher average dN/dS ratio between the five classes of alpha tubulin copies than within them, and a significantly higher dN/dS ratio within classes 2, 3, 4 and 5 than within class 1 (model 3 in Table 3, AIC = 29984 compared with 30004 for the next preferred branch-class model). This rejects the null model that all copies are under the same functional constraints, i.e. targeted by purifying selection towards the same amino acid sequence. Instead, it is consistent with each class being subjected to purifying selection, but that different classes have diverged in amino acid sequence more than expected based on the strength of purifying selection within each class. Within classes 2, 3 and 5, amino acid divergence occurs primarily between species, whereas in class 4 there are multiple copies within species that differ by 5.6 to 7% in amino acid sequence.

**Table 3:** **Comparison of alternative branch-class models (1, 2 and 3, Yang 1998**[45]**) and the branch-site model (4, Yang*et al.*2005**[46]**)**

**Model**	**LnL**	**Akaike Information Criterion**	**dN/dS ratios (± standard errors)**
1) ω_1_ = average dN/dS ratio across tree	−15025	30052	0.015±0.001
2) ω_1_ = dN/dS ratio within classes	−15000	30004	0.013 ±0.001
			0.078±0.011
ω_2_ = dN/dS ratio between classes			

3) ω_1_ = dN/dS ratio within class 1	−14989	29984	0.008±0.001
			0.016±0.001
			0.074±0.010
ω_2_ = dN/dS ratio within classes 2,3,4,5			
ω_3_ = dN/dS ratio between classes			
4) site class 0, ω_1_ = dN/dS of codons under equivalent purifying selection on both between class and within class branches. *p*_*1*_ = proportion of codons	−14945	29897	ω_1_ = 0.013±0.001
			*p*_*1*_ = 0.76±0.053
			ω_2_ = 1.00±0.011
site class 2a, ω_2_ = dN/dS of codons with higher dN/dS ratios on between class branches than on within class branches (with dN/dS ratio = ω_1_). *p*_*2*_ = proportion of codons			*p*_*2*_ = 0.24±0.053
(No codons were reconstructed to occupy site class 1 and 2b of model)			

The branch-site model confirmed these findings. An estimated 26% of codons belonged to a set having average dN/dS ratios of 0.013±0.001 on within class branches but of 1.0±0.01 on between class branches. The improved AIC of 29897 compared to the best branch-class model (AIC = 29984) indicates significant heterogeneity among codons in how they differ between branch classes. There is no evidence for positive selection at these sites according to the standard interpretation [46], because their average dN/dS ratio is optimized as 1.0, which equates to neutral evolution. There is evidence, however, that a quarter of codons that are under strong purifying selection within classes have diverged in amino acid sequence between classes at an average rate equivalent to synonymous sites. Twenty-two codons were shown by Bayes empirical Bayes analysis to have >0.95 posterior probability of belonging to this class (described further below in relation to protein structure and function).

### *Adineta ricciae* transcriptome search

Searching the *A. ricciae* transcriptome using BLAST yielded 11 hits for alpha tubulin that varied in length from 78 to 792 bases (median length = 354). Transcriptome sequences were all less than 3% divergent in nucleotide sequence from their nearest cloned and sequenced copy [see Additional file 4]. A distinct clade of four copies of class 1 alpha tubulin retrieved by cloning and sequencing was not represented by similar transcriptome sequence, possibly because those copies were not expressed in the experimental conditions used for isolating RNA for the transcriptome sequencing. One other copy, from class 5, also lacked a transcriptome match, but this copy was similar in sequence to other copies for which transcriptome matches were obtained. Together, we conclude that 5 out of 6 divergent copy types of *A. ricciae* alpha tubulin were expressed in laboratory conditions and 7 out of all 12 copies (including both divergent copy types and similar copies putatively maintained as templates for DNA repair) were found.

### Predicted protein chemistry of alpha tubulin classes

**Figure 5:**
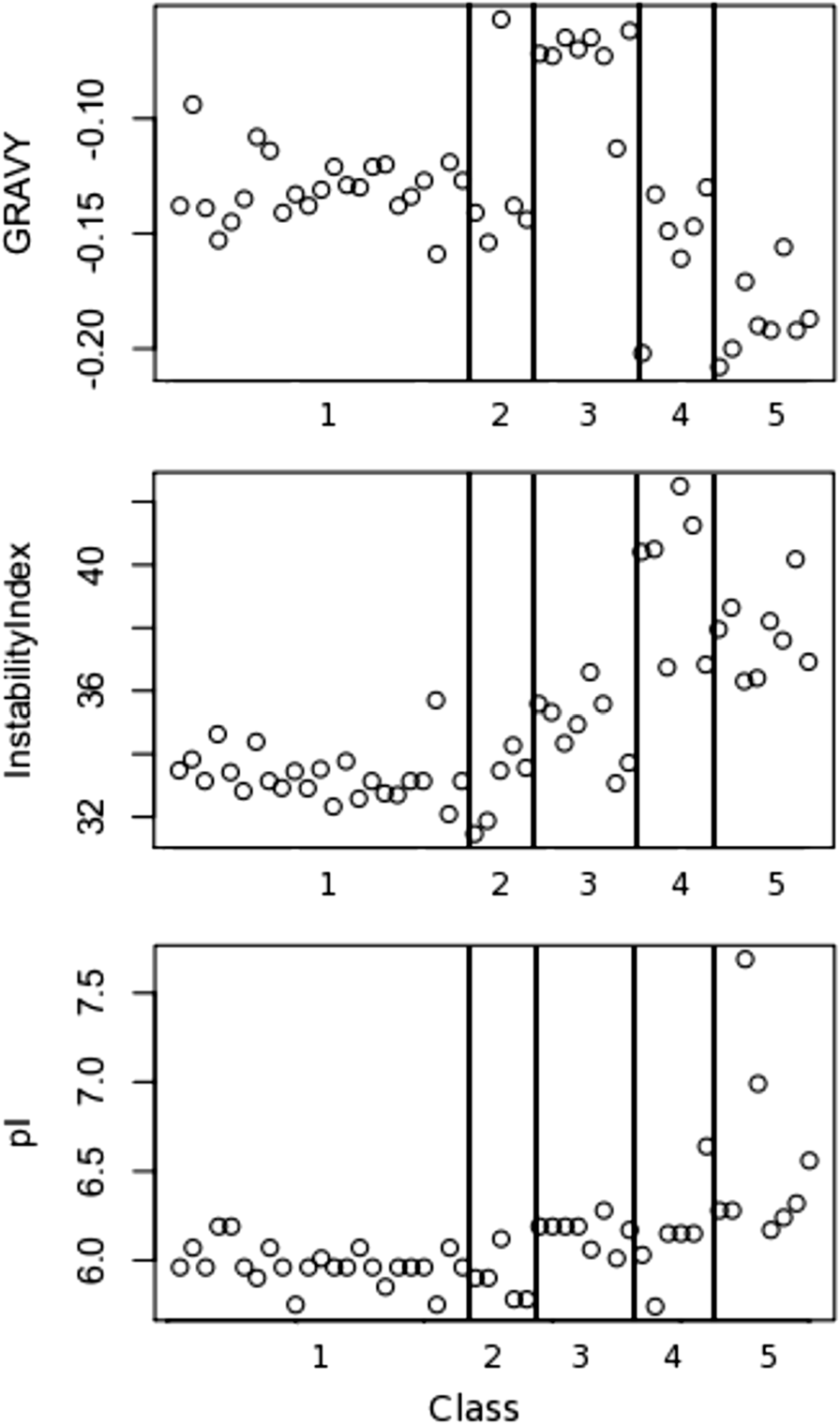
**Variation in indices of predicted protein biochemistry among all alpha tubulin copies, arranged by class.** Top = grand average of hydropathy (GRAVY), middle = Instability Index, bottom = isoelectric point (pI).

Classes based on intron structure differed significantly in predicted chemistry. The predictive discriminant analysis correctly identified copies of classes 3, 4 and 5 of alpha tubulin based on the three indices of protein chemistry (successfully identifying 100% of each class). Class 1 and 2 could be distinguished from the other classes but not from each other using the three indices of protein chemistry we considered (Figure 5). Class 1 and 2 copies are most stable and have the lowest isoelectric point; class 3 copies are significantly more hydrophobic (more positive GRAVY index) than copies of any other class (p < 0.0005 in comparison with all other classes, Tukey’s HSD); class 4 copies have the highest instability index (p < 0.0005 in comparison with classes 1–3, Tukey’s HSD); and class 5 copies are most hydrophilic (p < 0.005, Tukey’s HSD), have the highest isoelectric point (p < 0.05, Tukey’s HSD) and high instability (p < 0.0005 in comparison with classes 1–3, Tukey’s HSD). The same qualitative differences are found for the region of protein at the beta tubulin interface.

Mean MAPP scores of the 22 amino acid positions identified as significantly more divergent between classes than within them (BEB posterior probability > 0.95) were significantly higher than mean MAPP scores of a random selection of 22 codons across the alignment (10000 random samples, two-tailed p = 0.022), and also significantly higher than mean MAPP scores of other polymorphic sites (10000 random samples, two-tailed p = 0.0016).

### Amino acid divergence in relation to protein structure

Of the 22 amino acid positions evolving faster between classes than within them identified by the BEB analysis, none were in regions identified as functional [see Additional file 5]; there were significantly fewer of these codons in functional regions than would be expected by chance (10000 replicates, two-tailed p = 0.000). The GTP binding nonexchangeable site (codons 142 to 148), notably, was completely conserved across all copies in all species, and the conservation of the amino acids that interact with the nucleotides in GTP was 96%, 100% and 100% respectively. By contrast, the number of amino acid positions identified by the BEB analysis that fell within structural regions of the protein was not significantly different from random expectation (7 of these 22 codons, 10000 replicates, two-tailed p = 0.12).

## Discussion

Assuming that bdelloid rotifers are tetraploid and inherited alpha tubulin copies from a diploid common ancestor with monogononts, we expected bdelloids to have twice as many copies of alpha tubulin as diploid monogononts, or fewer than this if monogononts have copies with sex-specific functions that were lost in bdelloids. Instead, we found that alpha tubulin has undergone proliferation in bdelloid rotifers relative to the one or two copies found in monogonont rotifers, and beyond numbers expected solely based on degenerate tetraploidy. Each species contains between 11 and 13 copies that can be divided into five main classes supported both by exon phylogeny and intron structure. Roughly half the copies belong to a single class (class 1) and have amino acid sequences conserved both within the class and relative to copies present in monogonont rotifers. Within class 1, copies fall into four distinct groups (differing by more than 10% nucleotide sequence divergence, which we call copy types) in all four species. The remaining alpha tubulin copies belong to four classes that have diverged significantly in intron structure, amino acid sequence and, in the case of classes 3 to 5, in predicted biochemical properties. The four copy types within class 1 and the four divergent classes 2 to 5 together make eight divergent copy types per species. Because all five classes (including multiple copy types in class 1) are present in species from both sampled families of bdelloids, we propose that bdelloids inherited these eight divergent copy types from their common ancestor followed by the loss of some copy types in some species.

The repeated observation of pairs of similar sequences (<1.5% divergent) of a given copy type in the same species is consistent with there being pairs of copies maintained as templates for DNA repair through gene conversion, as proposed to occur between collinear pairs in other bdelloid genome regions [12]. An alternative explanation would be recent duplication of copies within species, but this would be unlikely to generate a widespread pattern of recent divergence between similar copies across classes, unless a similar event had happened independently in all four species. Cases in which only a single copy of a divergent copy type is present could be due to very recent conversion generating identical copies, or to the loss of some duplicate copies. Only in one case were more than two similar copies present within the same species (class 5 in *A. vaga*), and two of these sequences were just outside our error threshold, so perhaps reflect sequencing error.

Given the presence of paired copies within species’ copy types, we therefore propose that bdelloids inherited sixteen copies of alpha tubulin from their common ancestor: eight divergent copy types, each present as a pair of similar sequences. Some copies were later lost or homogenized in particular lineages leading to the observed numbers of copies in the sampled species. Alternative scenarios invoking duplication events since the species diverged cannot readily explain why all four species share these patterns, unless copies originating independently in separate species were either transferred horizontally to other species - which seem extremely unlikely, even with bdelloids’ known ability to take up foreign DNA - or had converged in both amino acid sequence and intron structure, again unlikely.

Our results provide robust evidence for numbers of copies and likely inheritance from their common ancestor, yet reconstructing the ancestry of alpha tubulin copies using phylogenetics proved challenging for two reasons. First, we detected major differences in base composition among sequences, which we attempted to deal with using models of composition heterogeneity in p4. Second, functional divergence between different classes has led to extreme rate heterogeneity and departures from the assumptions of neutral models of sequence evolution. For example, the relationships of class 1 copies relative to classes 2 to 5 were weakly supported and unstable among alternative reconstructions. The preferred tree using five base composition vectors had classes 4 and 5 as sister to one *A. vaga* class 1 copy type, which cannot reflect the true ancestry of the genes for reasons argued above (namely that horizontal transfer from one species followed by diversification across the other species seems highly unlikely). The amino acid tree does have class 1 copies as monophyletic and classes 2 to 5 copies as sister clade to them, which is consistent with inheritance of multiple copies from a common ancestor. However, the amino acid tree lacks resolution to infer ancestry among class 1 copies. Together, these problems prevented the use of formal genealogical methods for reconstructing patterns of gene duplication and loss (such as Notung 2.6 [58]). Also, the homogenizing effect of gene conversion is not taken into account in current approaches, and therefore the existence of recent pairs of copies within species would have been inferred as multiple duplications occurring in each species, rather than ancestrally inherited copies maintained by gene conversion (consistent with existing knowledge of bdelloid genomes). Our dataset presents a useful test case for developers of more complex models of sequence evolution.

Most monogonont species had one copy of alpha tubulin, which likely reflects a pair of identical alleles in these sexually reproducing diploids. This low copy number eliminates the possibility of sex-specific roles for alpha tubulin in most monogononts or that multiple copies in bdelloids arose from formerly sex-specific copies in their sexual ancestor. Assuming that the background level of ploidy in bdelloids conforms to the tetraploidy as demonstrated for two separate genome regions and in both bdelloid families we studied here [10], then if the ancestor contained four copies of alpha tubulin (one pair of alleles in the sexually reproducing ancestor duplicated – by either genome duplication or hybridization - on the origin of tetraploidy), a further two rounds of duplication of all genes are required to explain our findings. One possible mechanism would be tandem duplication, possibly facilitated by DNA breakage and repair during desiccation. Tandem duplication has been observed for alpha tubulin in species ranging from *Trypanosoma brucei*[59] to *Zea mays*[60]. If the ancestral sexual progenitor of bdelloids had two distinct copies of alpha tubulin, as found in the *B. plicatilis* transcriptome, then only one round of additional duplication events would be needed to explain our findings.

Why might bdelloids have so many copies of alpha tubulin? First, multiple identical copies might provide redundancy and increase the rate of production of proteins. Gene duplicates have been shown to provide robustness against null mutations in *Saccharomyces cerevisiae*[61]. Second, different copies might be expressed in different tissues or at different developmental stages. For example, *Bombyx mori* and *Drosophila melanogaster* alpha tubulin isotypes fall into classes that are ubiquitously expressed throughout the body at all life stages, classes that are expressed only at certain stages of the life-cycle, and classes expressed only in certain tissues, such as the testes or ovaries [62]. Third, different copies might have specialist functions in different cellular processes or locations. Modifications in amino acid sequence can result in changes in the stability and kinetics of microtubule assemblies and in the binding of different microtubule associated proteins [63]. Fourth, different copies might provide robustness against environmental variation, by maintaining microtubule function under extreme environmental conditions. For example nine copies of alpha tubulin expressed in brain tissue of the Antarctic fish *Notothenia coriiceps* vary in their stability and polymerization rates at extreme temperatures [21].

Which of these mechanisms might explain alpha tubulin proliferation in bdelloids? Class 1 copies have conserved protein structure and predicted biochemical properties shared with alpha tubulin copies in monogononts. Yet, each bdelloid species has four divergent types of this class of copies, plus additional pairs of similar copies. Interestingly, only two of the six class 1 copies were recovered in the transcriptome of *A. ricciae* pooled from several laboratory cultures both in hydrated and desiccated conditions. The missing copies might be expressed in small amounts or only in life-stages (e.g. eggs) or particular environments (e.g. rehydrating adults) that were not sampled in the experiments. Because no biochemical differences were detected among class 1 copies, these copies might be truly functionally redundant. Functional redundancy of housekeeping genes might provide extra protection in the face of DNA breakage by increasing the probability that at least one copy still functions after desiccation. Alternatively, multiple copies might provide the machinery to enhance rapid production of protein during rehydration. In *Arabidopsis thaliana* alpha tubulin is up-regulated upon rehydration after a period of dehydration [25]. Tubulin has also been shown to form aggregates and dense networks in *Brassica napus* seedlings during dehydration [64]. It is therefore possible that having multiple functionally redundant tubulin copies might enable rapid up-regulation of tubulin expression at important times.

In contrast, the remaining classes are divergent in amino acid sequence, and those present in *A. ricciae* were all recovered in the transcriptome and hence had been expressed under laboratory conditions. There was significant evidence for excess amino acid divergence between classes relative to the strong purifying selection within them. This is consistent with a standard model of functional specialization after gene duplication in which neo- or sub- functionalized copies evolve, perhaps following a period of relaxed selection, and are then subject to purifying selection. None of the amino acid positions identified as significantly divergent between classes are within regions identified a priori as functionally important for alpha tubulin, and very few are in structural regions, indicating that selection has not acted to alter fundamentally the functioning of the copies as microtubule proteins. These positions did, however, have significantly higher MAPP scores than random selections of other polymorphic codons, which indicates that they are predicted to have larger consequences for protein function than randomly selected amino acid positions. This finding adds further evidence that divergence at these sites is associated with functional divergence between classes. Class 2 alpha tubulin copies differed in amino acid sequence but have the same predicted chemistry as class 1 for the parameters examined here. However, this does not preclude a different function, as small amino acid changes can alter the function of tubulin [15]. Classes 3 to 5 have diverged significantly both in amino acid sequence and in predicted protein chemistry, and are therefore likely to play different roles.

Increased hydrophobicity of the domain that interacts with beta tubulin is predicted to increase microtubule stability [22]. Although there was no evidence of excess amino acid divergence in this region, contact surface hydrophobicity did differ significantly between classes and class 3 copies are predicted to form the most stable microtubule arrays and class 5 the least. Amino acid variation can also be involved in sub-cellular localization and cellular function. Variation in isoelectric point has been noted in tubulin copies with different sub-cellular distributions [53], so the significantly higher isoelectric point in class 5 relative to the others might point to a variation in sub-cellular localization or function between classes. Future experiments are needed to test these alternative hypotheses for the function of divergent copies.

Another striking difference between classes was in intron structure. In contrast to the lack of introns in monogonont alpha tubulin, many introns are present and their number and location varies markedly between classes. This finding matches broader evidence that intron loss and gain occurs at a significantly faster rate between paralogs than between orthologs [65,66]. Genetic manipulation experiments in rice and yeast have shown that introns influence the timing and cellular location of expression of alpha tubulin [67,68]. Such experiments are not feasible at present in bdelloid rotifers, for which no method of genetic modification is currently available. However, the significant pattern of divergence of intron structure between classes, and conservation within classes, adds further evidence for functional divergence among classes.

## Conclusions

Alpha tubulin has expanded into multiple copies in bdelloid rotifers compared to bdelloids’ nearest relatives, the monogonont rotifers. The copies differ in predicted functions: some provide redundancy for core functions similar to those in monogononts, whereas others have diverged in amino acid sequence and in predicted chemistry. Functional specialization of multiple copies might provide phenotypic flexibility to allow bdelloids to thrive in a range of different environments, which has been argued as a potential mechanism to compensate for their presumed slow rates of genetic adaptation [9,69]. However, expansion of gene families potentially reduces the potential efficiency of selection in bdelloid rotifers even further, by increasing the number of linked genes and the potential for interference among clones harboring alternative beneficial mutations [70]. Future work needs to link characterization of gene families like our study with understanding of the functional consequences of genetic variants for bdelloids in natural environments.

## Abbreviations

AIC, Akaike Information Criterion; aLRT, Approximate likelihood ratio test; BEB, Bayes empirical Bayes; BLAST, Basic Local Alignment Search Tool; dN/dS, Ratio (ratio of the rates of non-synonymous and synonymous substitutions); EST, Expressed sequence tag; G, Gamma-distributed rate variation; GRAVY Index, the grand average of hydropathy index; GTR, General time-reversible; I, Proportion of invariable sites; LEA, Late Embryogenesis Abundant; MAPP, Multivariate analysis of protein polymorphism; MAPs, Microtubule associated proteins; MCMC, Markov Chain Monte Carlo; NDCH, Node-discrete composition heterogeneity; pI, Isoelectric point; pp, Posterior probability.

## Competing interests

The authors declare that they have no competing interests.

## Authors’ contributions

IE, TB, DF, EH, CB and AT wrote manuscript. IE, EF, DF and EH performed the labwork. IE and TB analysed the data. CB, AC, GM and AT provided the draft transcriptome. All authors read and approved the final manuscript.

## Supplementary Material

Additional file 1 **Average GC content at the third codon position for sequences belonging to each species. BP=*Brachionus plicatilis* (monogonont); PF =*P. flaviceps*; AV=*A. vaga*; AR=*A. ricciae*; MQ=*M. quadricornifera*.** Kruskal-Wallis test for variation among species: chi-squared = 41.9, df = 3, p<0.0001. Click here for file

Additional file 2 **Model fit comparisons. Model fit comparisons.** Log marginal likelihood estimated from posterior samples using Equation (16) in Newton and Raftery (1994) implemented using func.newtonRaftery94_eqn16 [37] in p4 (v0.88; [34]). Click here for file

Additional file 3 **Alpha tubulin gene tree based on exon nucleotides including all cloned copies from all bdelloid species sequenced.** aLRT support values are shown and the scale bar is in units of substitutions per site. Click here for file

Additional file 4 **Alpha tubulin gene tree for*Adineta ricciae*including cloned and sequenced copies and copies found in the transcriptome.** Alpha tubulin gene tree for *Adineta ricciae* including both cloned and sequenced copies (named ArCopy1 etc.) and the copies found by searching the transcriptome using BLASTN (each named E_Locus followed by a number). Click here for file

Additional file 5 **Average dN/dS (Y-axis) obtained by sliding window analysis of the alpha tubulin alignment across classes.** Window size = 15, step size = 5. Colours along the x-axis show functional and structural regions: red = alpha helix; blue = beta sheet; green = functional regions (P = interacts with phosphate, ribose = interacts with ribose, others are annotated with their functions). Arrows = codons identified using Bayes empirical Bayes as experiencing divergent selection (position in alignment: 17, 19, 21, 22, 23, 31, 41, 71, 72, 104, 112, 127, 145, 146, 269, 272, 274, 276, 286, 317, 348, 355). Click here for file
