# Supplementary figures and images for: Multiple functionally divergent and conserved copies of alpha tubulin in bdelloid rotifers
Source: BMC Evol Biol. 2012 Aug 17;12:148. doi: 10.1186/1471-2148-12-148 (PMC3464624; doi:10.1186/1471-2148-12-148)

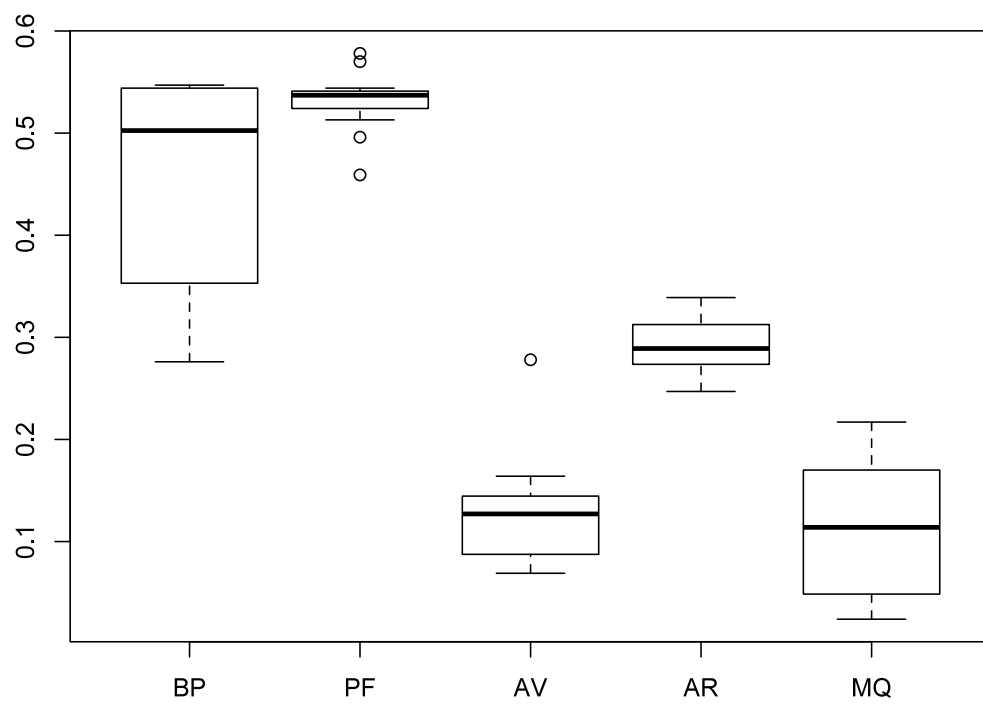

Supplement: Additional file 1 — Average GC content at the third codon position for sequences belonging to each species. BP=Brachionus plicatilis (monogonont); PF =P. flaviceps; AV=A. vaga; AR=A. ricciae; MQ=M. quadricornifera. Kruskal-Wallis test for variation among species: chi-squared = 41.9, df = 3, p<0.0001. [file 1471-2148-12-148-S1.pdf]

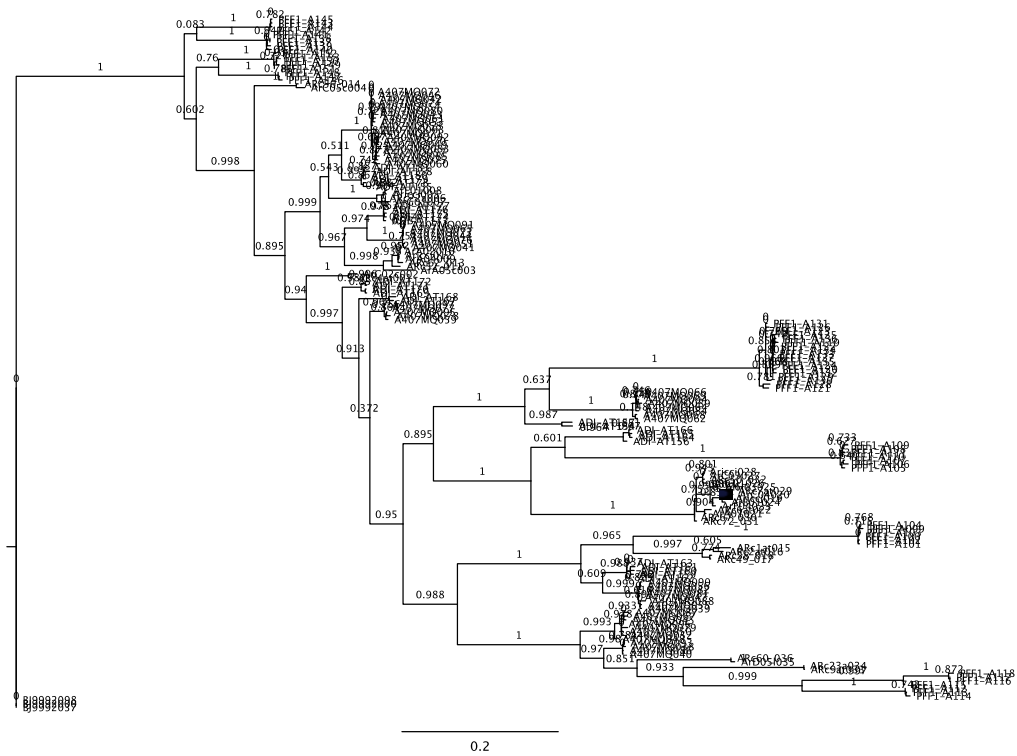

Supplement: Additional file 3 — Alpha tubulin gene tree based on exon nucleotides including all cloned copies from all bdelloid species sequenced. aLRT support values are shown and the scale bar is in units of substitutions per site. [file 1471-2148-12-148-S3.pdf]

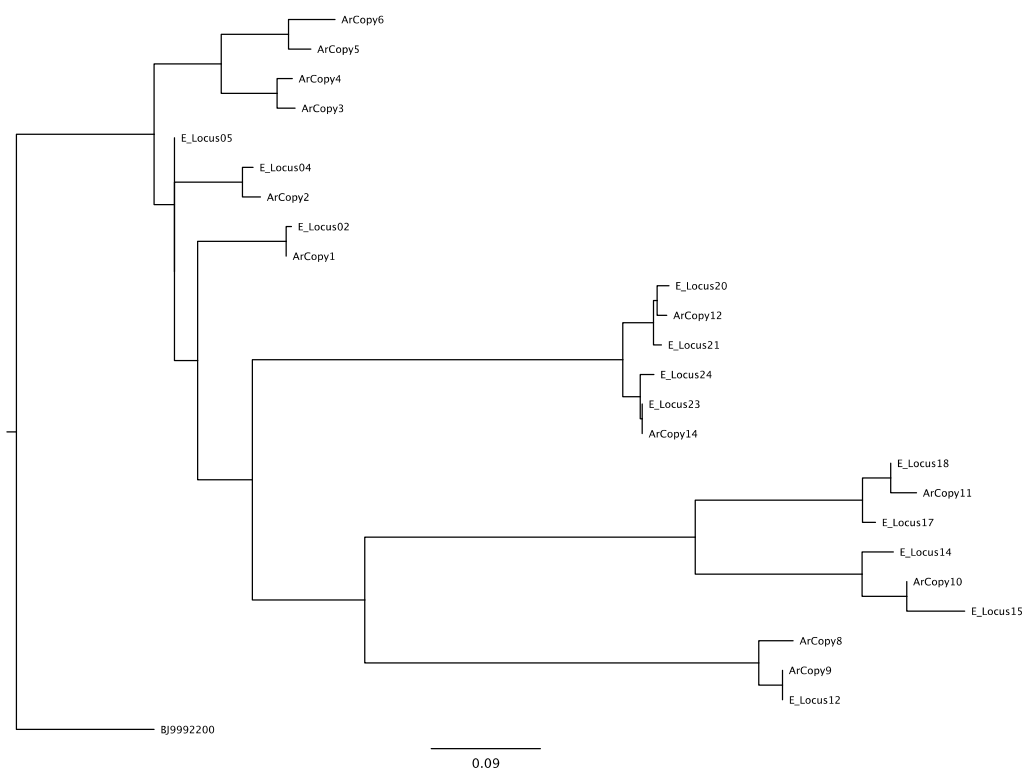

Supplement: Additional file 4 — Alpha tubulin gene tree forAdineta ricciaeincluding cloned and sequenced copies and copies found in the transcriptome. Alpha tubulin gene tree for Adineta ricciae including both cloned and sequenced copies (named ArCopy1 etc.) and the copies found by searching the transcriptome using BLASTN (each named E_Locus followed by a number). [file 1471-2148-12-148-S4.pdf]

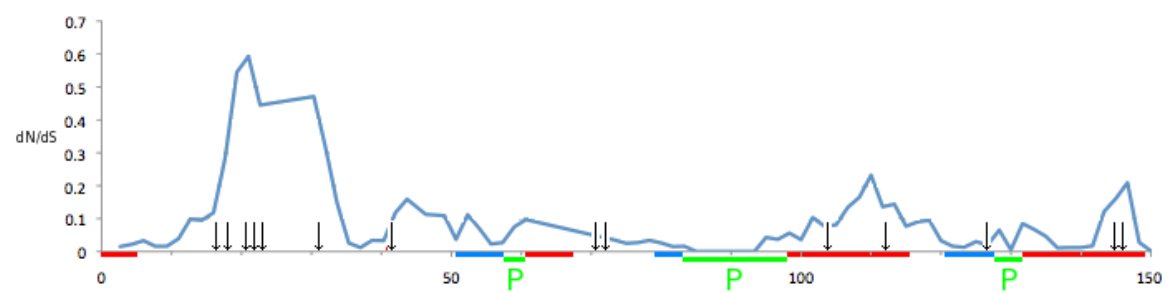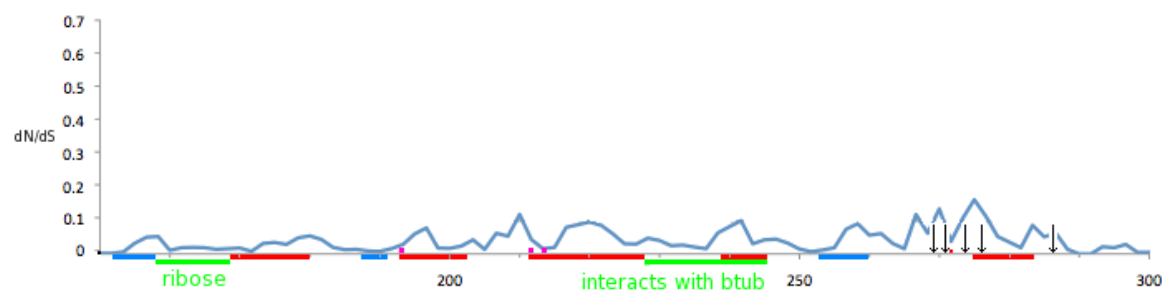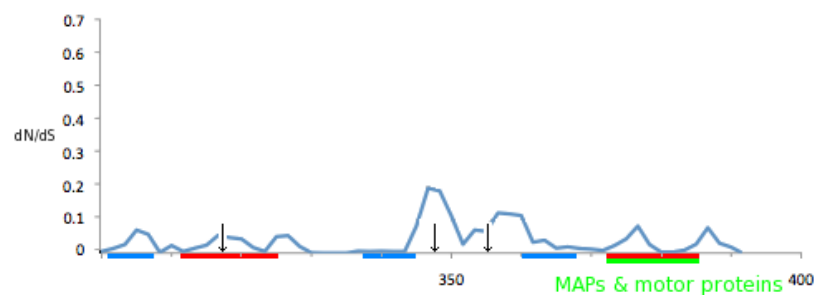

Supplement: Additional file 5 — Average dN/dS (Y-axis) obtained by sliding window analysis of the alpha tubulin alignment across classes. Window size = 15, step size = 5. Colours along the x-axis show functional and structural regions: red = alpha helix; blue = beta sheet; green = functional regions (P = interacts with phosphate, ribose = interacts with ribose, others are annotated with their functions). Arrows = codons identified using Bayes empirical Bayes as experiencing divergent selection (position in alignment: 17, 19, 21, 22, 23, 31, 41, 71, 72, 104, 112, 127, 145, 146, 269, 272, 274, 276, 286, 317, 348, 355). [file 1471-2148-12-148-S5.pdf]
