# Supplementary material for: Multiple functionally divergent and conserved copies of alpha tubulin in bdelloid rotifers
Source: BMC Evol Biol. 2012 Aug 17;12:148. doi: 10.1186/1471-2148-12-148 (PMC3464624; doi:10.1186/1471-2148-12-148)
Supplement: Additional file 2 — Model fit comparisons. Model fit comparisons. Log marginal likelihood estimated from posterior samples using Equation (16) in Newton and Raftery (1994) implemented using func.newtonRaftery94_eqn16 [37] in p4 (v0.88; [34]). [file 1471-2148-12-148-S2.pdf]

Model	LogMarginalLikelihood	Bayes Factor	$\log_e$(BF)
**GTR+I+G**	-17985.85	-	**-**
**NDCH(2)**	-17328.64	657	6.49
**NDCH(5)**	-17180.55	148.09	4.998
